# Biomechanical Evaluation of a Hybrid Fixation Strategy: Enhancing Intramedullary Nail Stability With a Trochanteric Stabilization Plate in Unstable Intertrochanteric Fractures With Lateral Wall Collapse

**DOI:** 10.7759/cureus.111635

**Published:** 2026-06-27

**Authors:** Nikhilesh Das, Nithin Shajeendran, Swathy G Nurani, Pranav Thaker, Nikunj Patel, Sudipto Mukherjee

**Affiliations:** 1 Department of Orthopaedics, Peerless Hospital and B.K. Roy Research Centre, Kolkata, IND; 2 Department of Design and Development, Miraclus Orthotech Pvt. Ltd., Vavdi, IND

**Keywords:** biomechanical testing, computational modelling, finite element analysis, hybrid fixation, intertrochanteric fracture

## Abstract

Introduction

Unstable reverse oblique intertrochanteric fractures with lateral wall collapse present a unique fixation challenge due to the absence of a lateral buttress. When treated with standard implants, this structural deficit frequently leads to construct failure through varus malunion and medialization of the femoral shaft. A device developed to provide rigidity for the maintenance of fracture reduction would bridge this gap. This study evaluates the biomechanical performance of a hybrid fixation strategy utilizing a Trochanteric Stabilization Plate for Nails (TSPN) in combination with an intramedullary nail (IMN) for such fractures using finite element analysis (FEA) and mechanical testing.

Methods

The study compared standard IMN fixation to a hybrid IMN-TSPN construct by simulating fracture scenarios in a validated femur model and performing mechanical tests on composite bone specimens (total n = 10; n = 5 per group). Finite element analysis quantified computational deformation, stress distribution, and elastic strain, while experimental physical tests measured construct stiffness and failure load under static axial compression. Statistical significance for experimental outcomes was evaluated using the non-parametric Mann-Whitney U test with a Bonferroni correction.

Results

The IMN-TSPN hybrid construct demonstrated a significant 1.85-fold increase in experimental structural rigidity (p = 0.008) and higher physical failure thresholds compared to IMN alone. Computationally, TSPN augmentation substantially reduced maximum fracture-gap displacement, peak elastic strain, and peak von Mises stress. The TSPN effectively redistributed loading and minimized total construct deformation.

Conclusion

In a composite bone model under static axial loading, augmenting an IMN with a TSPN significantly increased construct stiffness and reduced deformation compared to IMN alone. These findings suggest a biomechanical advantage that warrants further testing in clinically representative, dynamic models.

## Introduction

**Figure 1:**
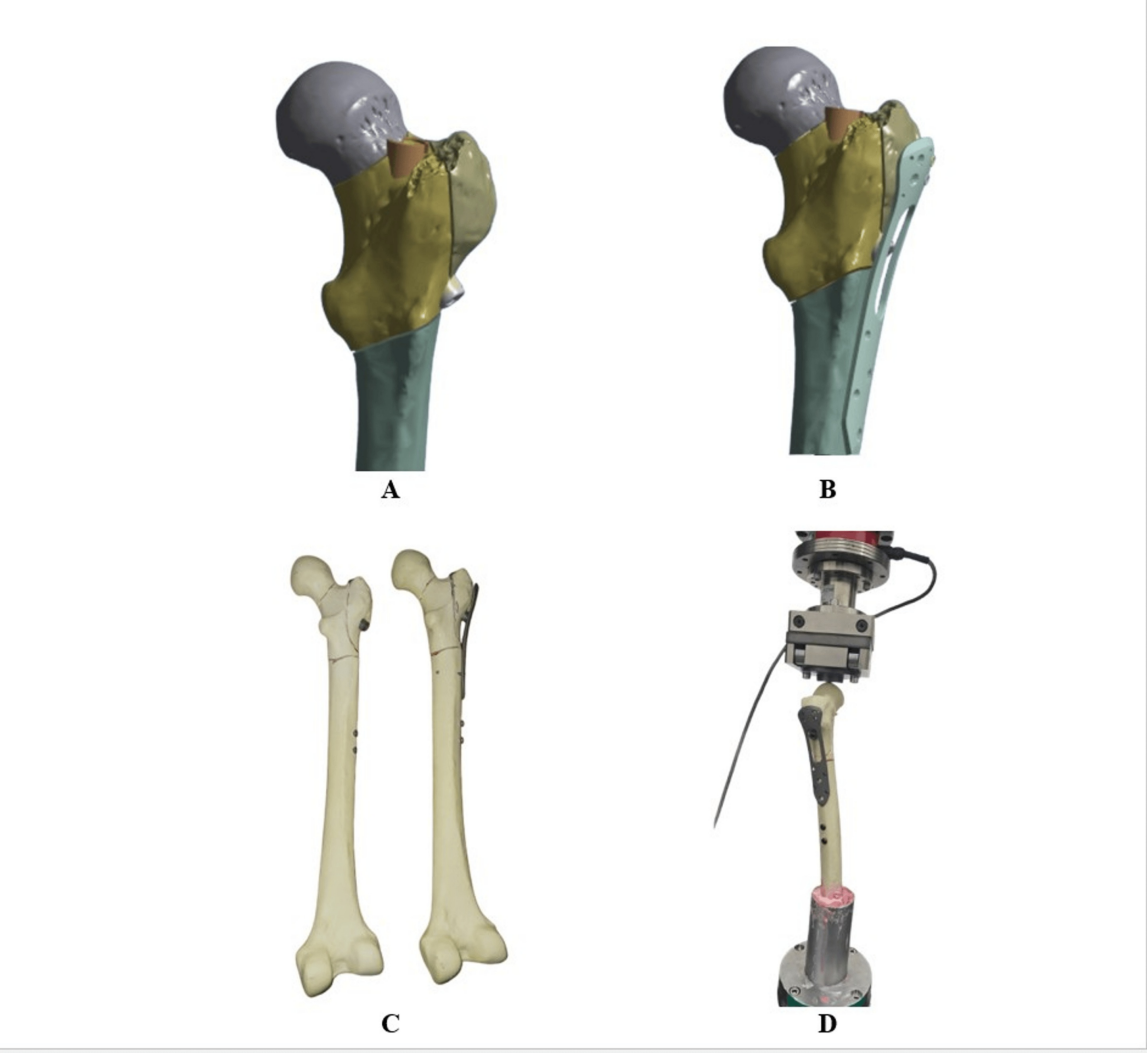
Experimental design. (A) FE bone model with an AO/OTA 31-A3 class fracture and intramedullary nail fixation. (B) FE bone model with an AO/OTA 31-A3 class fracture, intramedullary nail, and Trochanteric Stabilization Plate for Nails (TSPN). (C) Experimental models with an intramedullary nail and a combination of an intramedullary nail and TSPN. (D) Testing apparatus used.

Intertrochanteric hip fractures are among the most common injuries in orthopedic trauma in the elderly population, accounting for approximately 55% of proximal femoral fractures [1]. With global hip fracture incidences in adults over 50 projected to reach 4.5 million by 2050 [2], the burden is set to grow as populations age. One-year mortality following surgical management is estimated to be between 15% and 30%, and many survivors experience a permanent decline in mobility and independence [3], underscoring the urgency of effective surgical fixation. Among these fractures, the AO/OTA 31-A3 pattern presents a particular surgical challenge, as the inherently incompetent lateral wall eliminates the critical lateral buttress. When treated with standard implants, this structural deficit frequently leads to construct failure through lag screw cut-out, varus malunion, and medialization of the femoral shaft (Figure 1) [4]. Surgical fixation with intramedullary nails (IMNs) is the current standard of care for these fractures [5,6]; however, probabilistic modeling demonstrates that construct performance is highly sensitive to variations in bone mineral density and physiological loading [7], often failing to provide sufficient stabilization in high-grade fractures. The primary mechanical weakness of intramedullary nails in A3-type fractures is the lack of lateral support, which allows the proximal fragment to collapse. Although comparative studies indicate that nails offer superior axial strength compared to extramedullary plates, standalone plates provide specific advantages in terms of rotational stability [8,9].

To bridge this gap, a hybrid fixation strategy utilizing a Trochanteric Stabilization Plate for Nails (TSPN) alongside an IMN has been developed. The TSPN is designed to act as a lateral shield, containing the greater trochanter and providing the necessary rigidity to maintain fracture reduction during weight-bearing activities.

This study utilizes computational finite element analysis (FEA) to evaluate the biomechanical synergy of this hybrid construct [10,11]. By simulating an AO/OTA 31-A3 fracture in a validated femur model, we compared the performance of a standard IMN alone against an IMN-TSPN construct. Both models were subjected to a progressive loading protocol ranging from 100 N to 2400 N to simulate a variety of physiological conditions. The primary objectives were to quantify total deformation, von Mises stress distribution, and safety factors. We hypothesized that while a nail alone may suffice for stable patterns, the addition of a TSPN significantly enhances construct rigidity and the safety factor, ensuring superior fracture reduction and fixation longevity in high-grade, unstable injuries.

## Materials and methods

This study employed a two-phase approach integrating computational FEA with experimental biomechanical validation using composite femoral bone models. Two fixation constructs were evaluated for the management of unstable AO/OTA 31-A3 intertrochanteric fractures: (1) a proximal femur nail (PFN) (intramedullary nail) used in isolation and (2) a PFN augmented with a TSPN (Figure 1). 

Finite element analysis

A three-dimensional model of an adult femur was developed in SolidWorks (2024, Dassault Systèmes, Vélizy-Villacoublay, France), with geometry and dimensions conforming to the Synbone composite femoral model (#LS2162, Synbone AG, Zizers, Switzerland), ensuring correspondence between the computational and experimental specimens. An AO/OTA 31-A3 fracture pattern was simulated by introducing a comminuted intertrochanteric osteotomy with posteromedial comminution, consistent with the clinical definition of an unstable intertrochanteric fracture. Three-dimensional models of the IMN and TSPN, from Miraclus Orthotech Pvt. Ltd., were similarly developed in SolidWorks, and virtual implantation was performed anatomically with appropriate proximal and distal fixation, replicating standard intraoperative positioning (Figure 1).

A three-dimensional tetrahedral mesh was generated across all model components. All constituent materials were assumed to be homogeneous, isotropic, and linearly elastic [12]. Material properties for cortical bone, cancellous bone, and titanium alloy were assigned in accordance with the known mechanical characteristics of the composite model and standard titanium alloy implant specifications, as summarized in Table 1. To ensure the accuracy and reliability of the computational results, a mesh convergence study was performed using 10-node higher-order tetrahedral elements. A global element size of 2.0 mm was applied, with localized mesh refinement to 0.5 mm at the complex geometric curves and bone-implant contact interfaces. Mesh convergence was achieved when the difference in peak von Mises stress between successive refinements was less than 5%. 

**Table 1:** Material properties assigned in the finite element model.

Property	Unit	Cortical Bone	Cancellous Bone	Titanium Implants
Young's modulus	Pascal	18.5×10^9^	1.5×10^9^	112×10^9^
Poisson's ratio	-	0.3	0.3	0.33
Density	kg/m^3^	1900	500	4460
Yield strength	Pa	92.5×10^6^	6.5×10^6^	962×10^6^
Ultimate tensile strength	Pa	112.5×10^6^	18×10^6^	1032×10^6^

Static axial compressive loads of 600, 800, 1000, 1200, and 2400 N were applied along the femoral mechanical axis to simulate progressive physiological weight-bearing. The maximum load of 2400 N was selected to represent hip joint reaction forces up to approximately three times the body weight of a standard 80 kg (approximately 800 N) adult patient. Constructs were evaluated with respect to resultant deformation, von Mises stress distribution, and elastic strain. Bonded contact was defined at all bone-implant interfaces to simulate an idealized, maximally rigid scenario and to strictly evaluate the baseline comparative load-sharing mechanics between the two constructs [13].

Mechanical testing

The distal femur of each construct was rigidly potted and secured within a custom-fabricated alignment jig to maintain a consistent loading axis throughout testing, as illustrated in Figure 1. Axial compressive loading was applied through the femoral head using a servo-hydraulic universal testing machine (Instron, Norwood, Massachusetts) operating under displacement control at a constant rate of 5 mm/min. All tests were conducted under quasi-static conditions.

Load-displacement data were continuously acquired at an appropriate sampling frequency throughout each test. Construct stiffness was determined from the slope of the linear elastic region of the load-displacement curve using linear regression. Ultimate failure load was defined as the peak load recorded immediately prior to a sudden, irrecoverable increase in displacement, indicative of fracture collapse, implant cut-out, or gross construct failure. Testing was continued until one of these failure criteria was met for each specimen.

Data from the experimental testing were expressed as mean ± standard deviation. A total sample size of 10 specimens was used, divided equally into two groups: an unplated control group (IMN-only, n = 5) and a plated experimental group (IMN+TSPN, n = 5). Due to the small sample size, which precluded assumptions of normality, nonparametric Mann-Whitney U tests were used to compare the experimentally derived total deformation and stiffness between the two physical constructs. Statistical analyses were performed using R Studio. To mitigate Type I error inflation from multiple comparisons, a Bonferroni correction was applied, setting the threshold for statistical significance at α = 0.025. Note that inferential statistics (p-values) were not applied to the deterministic computational outputs (FEA stress, strain, and computational deformation).

## Results

The FEA of the two models revealed clear and clinically meaningful differences between the IMN-only construct and the IMN augmented with the TSPN.

In the nail-only construct (n = 5), fracture-gap displacement increased markedly with load, from a mean of 1.38 mm at 600 N to a maximum of 5.52 mm (± 0.45) at 2400 N, indicating limited primary stability and substantial interfragmentary motion (IFM) under near-physiological loading [11].

By contrast, the plated computational model showed reduced displacement across the same loading sequence (0.75-2.99 mm), with the plated femur exhibiting approximately 46% less deformation at 2400 N. In the physical testing, these values corresponded to an increase in experimental specific stiffness from 434.58 N/mm (± 35.6) in the unplated group to 801.86 N/mm (± 52.3) in the plated group, representing a statistically significant 1.85-fold improvement in structural rigidity (p = 0.008).

**Figure 2:**
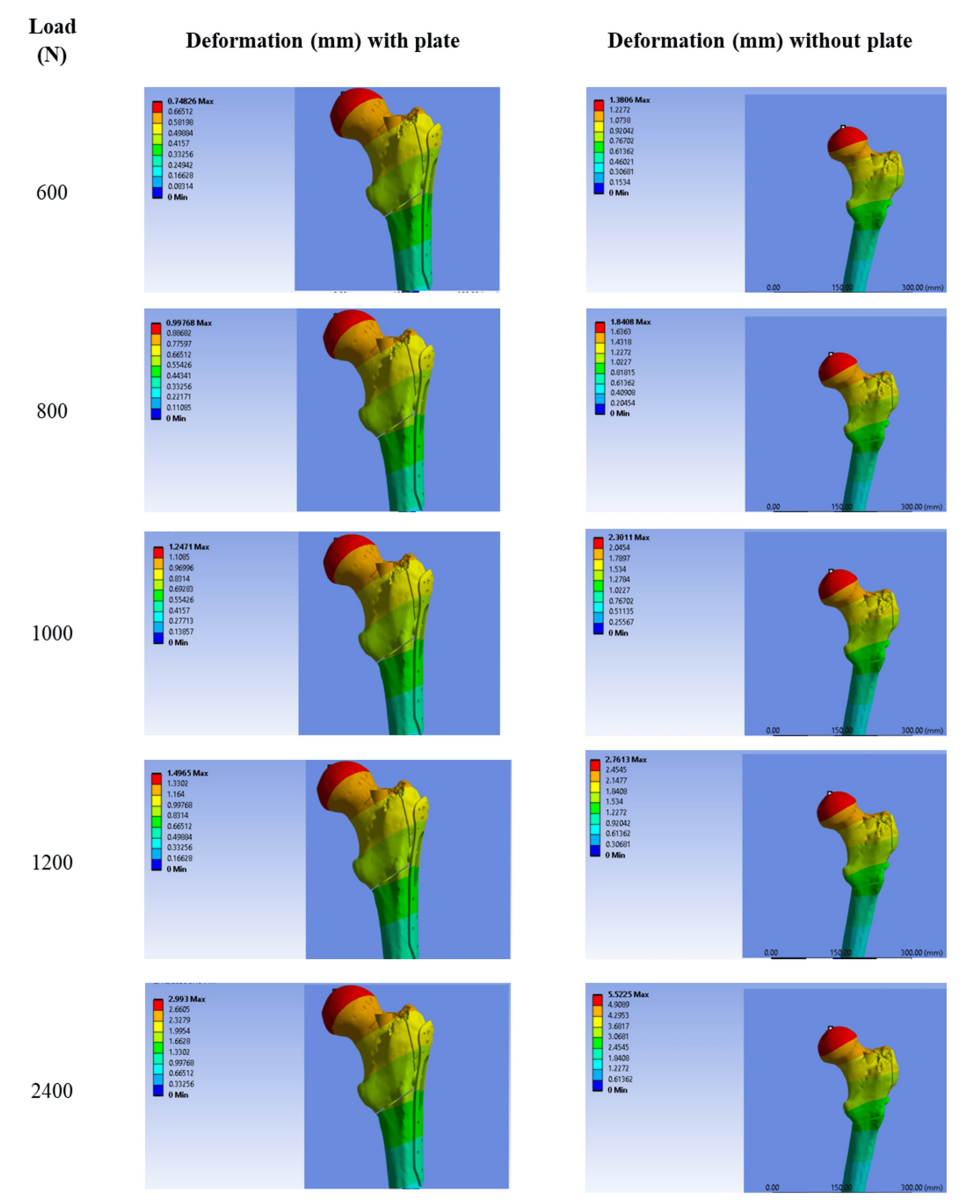
Total deformation (in mm) for the two constructs across load cases: with plate (left) and without plate (right).

Contour maps of total deformation and von Mises stress further demonstrate that the plate redistributes bending and axial loads, limiting proximal-distal segmental separation and reducing peak deformation at the fracture gap (Figure 2). 

**Figure 3:**
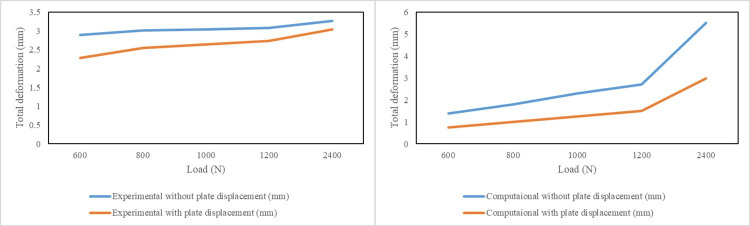
Maximum total deformation (mm) for the two constructs across different load cases: experimental results (left) and computational results (right).

Experimental biomechanical testing using a universal testing machine corroborated the computational trends. Load-displacement curves and measured failure loads confirmed that plate augmentation increased construct stiffness and increased the ultimate load before catastrophic displacement or collapse (Figure 3). 

**Figure 4:**
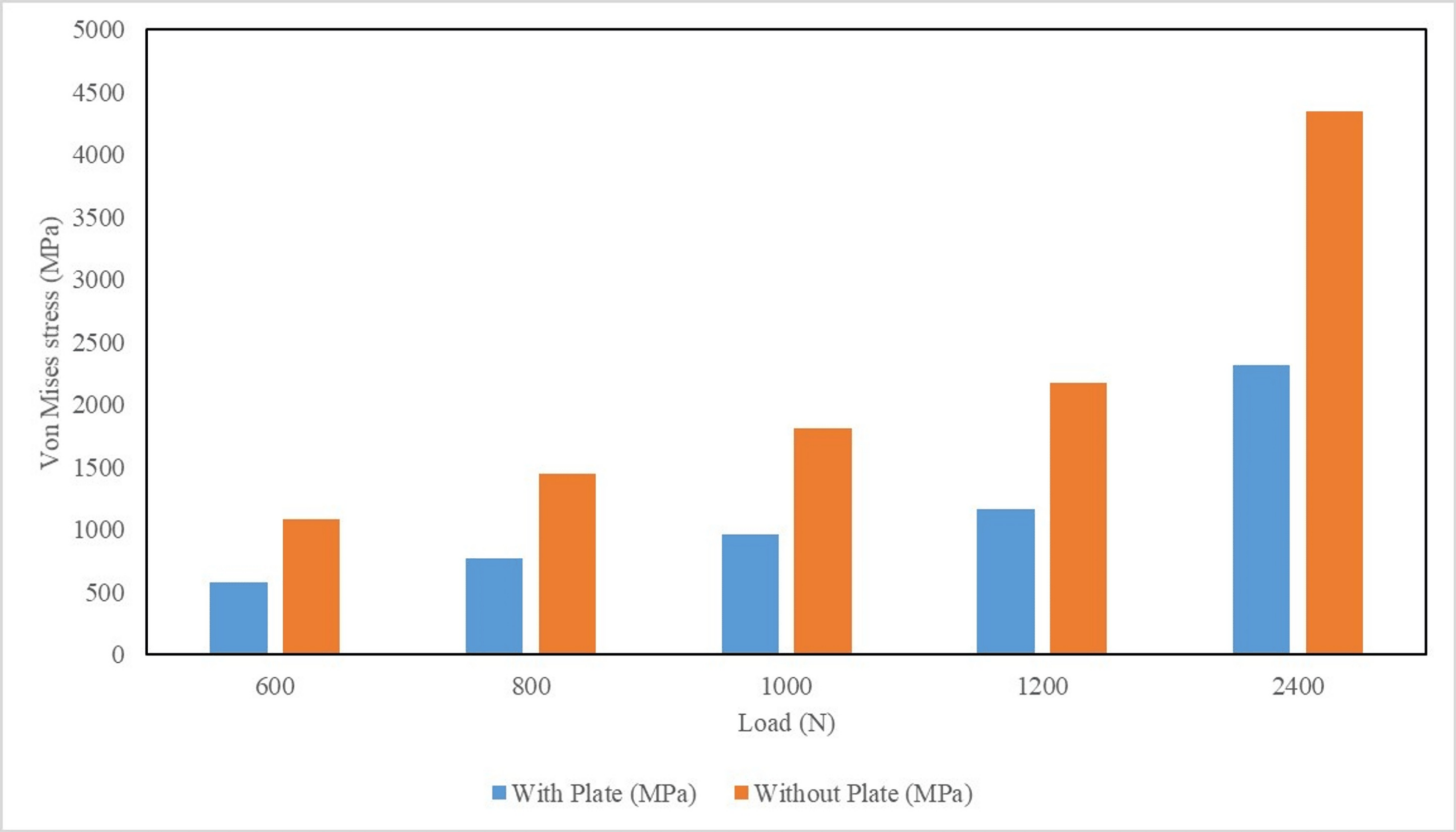
Von Mises stress (in MPa) calculated for the two constructs across load cases: with plate and without plate.

The von Mises stress analysis demonstrated a near-linear increase in peak stress with applied load for both constructs, but with markedly different magnitudes (Figure 4). In the IMN-only computational model, peak von Mises stresses rose from ≈1,100 MPa at 600 N to ≈4,300 MPa at 2,400 N. With the TSPN, peak von Mises stresses were reduced to ≈600 MPa at 600 N and ≈2,300 MPa at 2,400 N. It should be noted that these absolute peak values exceed reported titanium yield strengths (≈825-1,100 MPa) and represent computational singularities resulting from the bonded contact assumptions at the bone-implant interface. While the absolute peak values are artifactual, the relative comparative trend is robust: the TSPN offloads the fractured cortex by sharing axial and bending moments with the implant, achieving an approximate 46.5% reduction in peak local stress compared to the IMN alone. 

## Discussion

Mechanistically, the TSPN functions as a bridging tension band: it reduces tensile strain at the lateral cortex and converts disruptive tensile loads into compressive forces across the fracture interface [12]. By providing a lateral buttress, the plate offloads the fractured cortex and shares axial and bending moments with the intramedullary implant, thereby reducing the local stress burden at the fracture margins.

When interpreting the finite element analysis, it should be noted that the absolute peak stresses exceeded reported cortical bone strength (yield ≈115 MPa; ultimate ≈135 MPa) and, at the highest simulated peaks, approached or surpassed typical yield ranges for Ti-6Al-4V (≈825-1,100 MPa). However, such extreme local values represent mathematical artifacts of the computational model, specifically stress concentrations resulting from geometric singularities and the bonded contact assumptions [14,15]. Because bonded contacts simulate an idealized, maximally rigid scenario, they cannot reflect true frictional sliding. Despite these computational limitations, the comparative trend remains robust: TSPN augmentation significantly reduces peak stress relative to the unplated construct.

The elastic strain results corroborate the stress findings. Peak elastic strains in the unplated construct reached 4.75% under 2,400 N of load. Because the typical yield strain for cortical bone is approximately 1% to 2%, this extreme strain value computationally implies a high risk of localized bone failure or implant cut-out at the lateral cortex in a clinical setting. By contrast, the TSPN construct limited peak strain to 2.30%. According to interfragmentary strain theory, reducing extreme strain shifts the mechanical environment toward a window more classically associated with stable secondary healing (approximately 2-10% strain), minimizing the mechanical conditions that typically lead to construct collapse [16,17].

For unstable AO/OTA 31-A3 intertrochanteric fractures, these combined computational and experimental data indicate that while an IMN may provide acceptable load sharing in less severe patterns, augmentation with a TSPN substantially improves mechanical stability and increases structural safety margins under physiological loads [5]. Mechanically, the TSPN increases construct stiffness and limits total construct deformation under functional loads (e.g., ~1,200 N during gait and ~2,400 N during stumbling).

While absolute numeric peaks and biological extrapolations must be interpreted with caution, given the use of static loading and synthetic composite models, these findings demonstrate a clear biomechanical advantage for the hybrid construct. Clinically, these findings suggest that augmentation with a TSPN provides enhanced mechanical stability, which warrants further investigation in dynamic, clinically representative, and osteoporotic models to confirm its efficacy for early patient mobilization.

Study limitations

While this study provides valuable comparative insights, several significant limitations must be acknowledged. First, the sample size for the experimental testing was very small (n = 5 per group), which limits the statistical power and generalizability of the physical data. Second, the finite element analysis and mechanical testing utilized static loading protocols, which simplify the complex, dynamic, and cyclic fatigue forces experienced in vivo during activities of daily living. Third, the stabilizing contributions of surrounding soft tissues, musculature, and the joint capsule were not modeled, representing a worst-case mechanical scenario. Fourth, the computational model utilized bonded contact formulations to establish an idealized comparative baseline; this artificially rigidifies the construct and cannot accurately simulate frictional sliding or true interfragmentary motion present in the immediate postoperative environment. Consequently, the absolute FEA stress peaks represented mathematical singularities rather than physiological reality. Finally, the use of synthetic composite bones, while excellent for minimizing inter-specimen variability and exhibiting the assumed isotropic properties, represents a major oversimplification of living human bone, which is anisotropic, heterogeneous, and viscoelastic. Therefore, these models cannot account for biological healing processes, variations in patient anatomy, or conditions such as severe osteoporosis. As an in vitro and in silico evaluation, this study does not provide data on clinical patient outcomes such as union rates, pain, or complications.

## Conclusions

Utilizing a dual-methodology approach involving in vitro physical testing and in silico finite element analysis, this study evaluated the stabilization of unstable AO/OTA 31-A3 intertrochanteric fractures under static axial compression. The results demonstrate that augmenting a standard IMN with a TSPN acts as an effective lateral buttress. This hybrid construct significantly increased experimental construct stiffness and minimized computational fracture-gap deformation compared with an IMN used in isolation. By mitigating the structural deficits inherently associated with lateral wall collapse, the TSPN offloads adverse local stresses and provides a clear biomechanical advantage in a controlled laboratory setting. However, while these static, synthetic models establish foundational mechanical efficacy, they cannot fully replicate biological complexity. Consequently, these findings warrant further investigation utilizing dynamic cyclic loading, osteoporotic bone models, and subsequent clinical trials to determine the hybrid construct's impact on long-term patient outcomes and secondary fracture healing.
